# When Activator and Inhibitor of PPARα Do the Same: Consequence for Differentiation of Human Intestinal Cells

**DOI:** 10.3390/biomedicines9091255

**Published:** 2021-09-17

**Authors:** Katerina Cizkova, Tereza Foltynkova, Jiri Hanyk, Zbynek Kamencak, Zdenek Tauber

**Affiliations:** Department of Histology and Embryology, Faculty of Medicine and Dentistry, Palacky University, 77900 Olomouc, Czech Republic; katerina.cizkova@upol.cz (C.K.); tereza.foltynkova@upol.cz (F.T.); jiri.hanyk01@upol.cz (H.J.); zbynek.kamencak01@upol.cz (K.Z.)

**Keywords:** cell proliferation, cell differentiation, colorectal carcinoma, peroxisome-proliferator activated receptor

## Abstract

Peroxisome proliferator-activated receptor α (PPARα) is a ligand-dependent transcription factor that plays a role in various processes including differentiation of several cell types. We investigated the role of PPARα in the differentiation of intestinal cells using HT-29 and Caco2 cell lines as a model as well as human normal colon and colorectal carcinoma tissues. We detected a significant increase in PPARα expression in differentiated HT-29 cells as well as in normal surface colon epithelium where differentiated cells are localised. Thus, it seems that PPARα may play a role in differentiation of intestinal cells. Interestingly, we found that both PPARα activators (fenofibrate and WY-14643) as well as its inhibitor (GW6471) regulated proliferation and differentiation of HT-29 cells in vitro in the same way. Both compounds led to a decrease in proliferation accompanied by a significant increase in expression of villin, intestinal alkaline phosphatase (differentiation markers). Moreover, the same trend in villin expression was observed in Caco2 cells. Furthermore, villin expression was independent of subcellular localisation of PPARα. In addition, we found similar levels of PPARα expression in colorectal carcinomas in comparison to adjacent normal epithelium. All these findings support the hypothesis that differentiation of intestinal epithelium is PPARα-independent.

## 1. Introduction

Peroxisome proliferator-activated receptor α (PPARα) is a ligand-dependent transcription factor that belongs to the nuclear receptor superfamily. PPARα is activated by a wide range of both endogenous and exogenous ligands such as dietary fatty acids, eicosanoids, hypolipidaemic drugs, phthalates and pesticides [1,2]. After ligand binding, PPARs heterodimerise with retinoid X receptor, bind to peroxisome proliferator response elements (PPREs) and regulate the expression of a number of target genes [3]. PPARα regulates various cellular function including energy metabolism, oxidative stress, immune response, xenobiotic metabolism, cell proliferation, differentiation and carcinogenesis [1,2,3,4,5].

PPARα is molecular target of fibrates. Fibrates, such as fenofibrate, bezafibrate, clofibrate and gemfibrozil, are widely used drugs that reduce serum lipids, and they are the first choice for hypertriglyceridemia when a change in diet is not sufficient. These compounds are generally well-tolerated drugs with good safety profiles [6,7,8]. Other synthetic compounds that activate PPARα, such as WY-14643 (or pyrinixic acid), are not approved for clinical use. However, they are often used for research purposes [6]. Based on mouse models of human diseases, fenofibrate protects the intestine from colitis-induced permeability [9], and it could also have a possible therapeutic potential in Crohn’s disease [10]. Although there are studies suggesting that synthetic PPARα ligands could be useful for prevention and treatment of different cancers [3,11,12,13,14,15,16], there are also studies which have described an increase in cell proliferation after fibrates treatment [17,18,19]. Thus, further investigation of their potential use in cancer treatment is needed.

The activation of PPARα plays a role in the differentiation of various cell types. It is necessary for the differentiation of murine embryonic stem cells into cardiomyocytes in vitro [20]. It also leads to the promotion of adipocyte differentiation [21] as well as of angiogenic progenitor cell differentiation toward endothelial lineage [22]. It is known that activation of PPARα enhances cardiac progenitor differentiation [23]. Moreover, PPARα regulates myeloid lineage differentiation, but it negatively modulates the differentiation of bone marrow-derived progenitor cells [24]. In addition, activation of PPARα promotes osteogenic differentiation of MC3T3-E1 cells [25] and PPARα stimulates keratinocyte differentiation in mice and human skin explants [26].

Intestinal epithelium regenerates intensively during life. The undifferentiated cells are localised in the crypt area; during differentiation into absorptive and secretory cells, they migrate upward and are shed out at the villous tips [27]. Although PPARα is expressed in intestines very early in prenatal development, its role in intestinal cell differentiation is unknown. Previous studies have shown detection of PPARα expression in human intestines during prenatal development at mRNA [1] as well as protein levels [28,29]. PPARα mRNA level has been increased with gestational age in foetuses from 8 to 18 weeks of age [1]. PPARα has been weak in jejunum at 7 and 12 weeks and not detected at 16 weeks, while the expression in the ileum was moderate at 12 and 16 weeks and increased at 22 weeks. PPARα has been detected in colon at 8 weeks, but it has been decreased at 14 and 20 weeks [28]. The expression of PPARα also differs along the crypt–villous axis. Until the 11th week of prenatal development, expression has been stronger in the area of future crypts than in apical parts of villi. After this period, the expression along crypt–villous axis has been comparable [29]. PPARα is also expressed in postnatal life. In murine small intestine, the expression of PPARα has shown to increase according to the crypt–villous axis [30]. In humans, PPARα mRNA has been detected in normal colon tissue [31], although only a low expression of PPARα has been detected at the protein level by immunohistochemistry [4]. Moreover, in vitro differentiation of Caco2 cells in long-term culture was accompanied with an increase in PPARα expression [32].

The aim of this study was to investigate the possible role of PPARα in cell differentiation using HT-29 and Caco2 cells as a model. Under standard culture conditions, these cells do not differentiate, but they can be differentiated in vitro after sodium butyrate treatment (HT-29) or spontaneously in post-confluence culture conditions (Caco2) and then resemble human colon epithelium [33,34]. We examined the effect of PPARα activators fenofibrate and WY-14643 and PPARα inhibitor GW6471 on cell proliferation activity and expression of villin and intestinal alkaline phosphatase (as the markers of intestinal cell differentiation) as well as PPARα expression itself. Moreover, as carcinogenesis could be seen as result of the disruption of the normal differentiation process, the PPARα expression pattern in colorectal carcinoma and healthy margin tissues samples was also explored.

## 2. Material and Methods

### 2.1. Cell Culture and Treatment

Human colorectal tumour-derived cell lines HT-29 and Caco2 were obtained from American Type Culture Collection. The cell lines’ authentication via STR profiles was performed by the Department of Clinical Genetics, Palacky University, Olomouc. The cells were routinely cultured in DMEM (Sigma–Aldrich, St. Louis, USA, cat. no. D6171) supplemented with 10% (HT-29) and 15% (Caco2) FBS (HyClone, Marlborough, USA, cat. no. SV30160.03), penicillin (100 U/mL), and streptomycin (100 mg/L). Cells were incubated at 37 °C and 5% CO_2_ and passaged twice per week.

The whole experimental procedure is summarised in Supplementary Materials Figure S1. Undifferentiated cells from both cell lines were seeded and adhered overnight. The seeding density was dependent on the assay. For the proliferation assay and In-Cell ELISA, the cells were seeded in 96-well cultivation plates (TPP, cat. no. 92696) at a density of 10,000 cells/well (HT-29) and 7000 cells/well (Caco2). For immunocytochemistry and multiplex immunofluorescent staining, the HT-29 cells were seeded in 8-well cell culture slides (SPL Life Sciences, Naechon-Myeon, Korea, cat. no. 30108) at a density off 18,000 cells/well.

The next day after seeding, the cells were treated with PPARα activators (fenofibrate (Cayman Chemicals, Michigan, USA cat. no. 10005368) or WY-14643 (Sigma–Aldrich, St. Louis, USA, cat. no. C7081) and PPARα inhibitor (GW6471 (Cayman Chemicals, Michigan, USA, cat. no. 11697)) in the following concentrations: 25 μM and 150 μM (HT-29) or 200 μM (Caco2) fenofibrate, 25 μM and 200 μM WY-14643, and 1 μM and 10 μM GW6471. The undifferentiated control cells were treated with an appropriate concentration of DMSO. Then, the cells treated with PPARα ligands (or DMSO) were incubated for 72 h at 37 °C.

For obtaining differentiated cells, HT-29 cells were seeded, incubated overnight, and then treated with 5 mM sodium butyrate (NaBt; Sigma–Aldrich, St. Louis, USA, cat. no. B5887) for 72 h. For obtaining differentiated Caco2 cells, the cells were cultured for 14 days after reaching confluence. The growth medium was changed twice per week. After the differentiation process, the medium was changed and the cells were treated with PPARα ligands for 72 h, as mentioned above. The differentiated cells used as controls were treated by appropriate concentration of DMSO. The cells were not reseeded during the experiments.

### 2.2. Proliferation Assay

The effect of used concentrations of fenofibrate, WY-14643, and GW6471 on cell proliferation in both undifferentiated and differentiated cells was measured by the WST-1 proliferation test (Roche, cat. no. 11644807001) carried out according to the vendor’s protocol. After the incubation period with tested PPARα activators and inhibitor, WST-1 reagent was added and incubated for 60 min, (37 °C, 5% CO_2_). Then, the absorbance was measured by the microplate reader Power Wave XS (Bio-Tek, Winnoski, USA) at 450 nm. The WST-1 test was performed in three independent triplicates (*n* = 9).

### 2.3. In-Cell ELISA (ICE)

The changes in protein expression of known markers of intestinal differentiation, villin and intestinal alkaline phosphatase (IAP) as well as PPARα, itself, were investigated by the In-Cell ELISA colorimetric kit (ThermoScientific, Waltham, USA, cat. no. #62200). After the incubation period, the cells were washed with PBS and fixed with 4% paraformaldehyde for 10 min at RT. The procedure was performed according to the vendor’s protocol. The following rabbit polyclonal primary antibodies were used: villin (GeneTex, Hsinchu, Taiwan; cat. no. GTX110034) at a dilution of 1:1500; IAP (GeneTex, Hsinchu, Taiwan, cat. no. GTX112100) at a dilution of 1:500; PPARα (GeneTex, Hsinchu, Taiwan, cat. no. GTX28934) at a dilution of 1:1000. The antibody signals (measured as absorbance at 450 nm) were normalised to Janus green staining intensity (a mitochondrial dye; measured as absorbance at 615 nm) to account for cell number variation. The results are shown as relative expression (%) in comparison to appropriate control cells (100%). The absorbance was measured by microplate reader Power Wave XS (Bio-Tek, Winnoski, USA). The experiment was performed in three independent duplicates (*n* = 6).

### 2.4. Immunocytochemistry

The HT-29 cells were seeded in 8-well cell culture slides treated with 150 μM fenofibrate, 200 μM WY-14643, and 10 μM GW6471 as mentioned above and then fixed with 4% paraformaldehyde for 15 min. Before immunostaining, the cells were hydrated, permeabilised with 0.1% Triton-X for 15 min, and heat-induced antigen retrieval in citric buffer pH6 (120 °C, 15 min, Histos device) was performed. After that, the endogenous peroxidase activity was blocked by PolyDetector Peroxidase Blocker (Bio SB, part of the detection kit) for 5 min and cells were incubated 10 min with ProteinBlock (Dako, Glostrup, Denmark). The samples were incubated with PPARα primary antibody (GeneTex, Hsinchu, Taiwan, cat. no. GTX28934) at dilution 1:200 overnight at 4 °C. The reaction was visualised by Mouse/Rabbit PolyDetector DAB HRP Brown kit (Bio SB, Santa Barbara, USA, cat. no. BSB 0205). Tris buffer with TWEEN 20 (pH 7.6) was used for washing between the different steps. Nuclei were counterstained with haematoxylin, washed in tap water, dehydrated, and cover slipped. The staining results were evaluated as the % of cells from 5 independent fields of vision at a magnification of 400x.

### 2.5. Multiplex Immunofluorescence Staining

To confirm that the villin expression was independent of the subcellular localisation of PPARα, we used an Opal™ 4-Color Manual IHC Kit (Perkin Elmer, Walthem, USA, cat. no. NEL810001KT) according to the vendor’s protocol. The undifferentiated HT-29 cells were seeded in 8-well cell culture slides, adhered overnight, and treated with 150 μM fenofibrate and 10 μM GW6471 for 72 h. After that, the cells were fixed with 4% paraformaldehyde for 10 min at RT and were stained. The rabbit monoclonal primary antibody anti-villin (Abcam, Cambridge, UK, cat. no. ab130751) at a dilution of 1:100 and PPARα (GeneTex, Hsinchu, Taiwan, cat. no. GTX28934) at a dilution of 1:100 was used.

### 2.6. Oil Red O Staining and Quantification of Lipid Content

The cells were seeded in 8-well cell culture slides and adhered overnight. The next day, the undifferentiated cells were treated with 150 μM fenofibrate, 200 μM WY-14643, and 10 μM GW6471 and incubated for 72 h. The differentiated cells were pre-treated with 5 mM sodium butyrate for 72 h; after that, the medium was changed and the cells were treated with 150 μM fenofibrate, 200 μM WY-14643, and 10 μM GW6471 and incubated for 72 h. After the incubation period, the samples were washed with PBS and fixed with 4% paraformaldehyde for 10 min at RT. The cells were washed in 60% isopropyl alcohol and then stained with Oil Red O solution (0.3% Oil Red O (Sigma–Aldrich, St. Louis, USA; cat. no. O0625) in 60% isopropyl alcohol) for 45 min. Then, the slides were washed in 60% isopropyl alcohol followed with water. The cell nuclei were counterstained with haematoxylin and the slides were cover slipped in AquaTex mounting medium (Dako, Glostrup, Denmark, cat. no. S3025).

For quantification, the cells were seeded in 96-well plates as well as for ICE method mentioned above. After incubation, the cells were washed with PBS and fixed with 4% paraformaldehyde for 10 min at RT, then washed with 60% isopropyl alcohol. Then, 100 μL of Oil red per well were added and incubated for 45 min at RT. The plate was washed six times with deionised water. Next, 60% isopropyl alcohol was added for 10 min to elude the dye. The absorbance of extracted dye at 510 nm was measured by microplate reader Power Wave XS (Bio-Tek). The plates were washed and stained by Janus green (absorbance measured at 615 nm) to account for the cell number. The normalised absorbance A510/A615 was calculated and the results are shown as the mean ± SD (*n* = 12).

### 2.7. Immunohistochemical Detection of PPARα

Tissue samples of colorectal adenocarcinoma and adjacent normal colon tissue (i.e., both samples from one patient) were obtained from the archives of the Department of Clinical and Molecular Pathology, Faculty of Medicine and Dentistry, Palacky University, Olomouc. The total number of patients was 37 (26 males, 11 females; all patients were Caucasians). No patient obtained any anticancer treatment before surgery. The average age of the patients was 66.54 ± 11.30 years, median 69.00 years (males: average 65.77 ± 11.63, median 69.00 years; females: average 68.36 ± 10.80 years, median 70.00 years). The sample collection contained grade 1 (*n* = 9), grade 2 (*n* = 20), and grade 3 (*n* = 8) carcinomas. The basic patients´ characteristics (i.e., age, sex, grading, and TNM staging) are provided in Table S1. The use of all samples was approved by the Ethics Committee of the University Hospital Olomouc (protocol No. 134/14).

PPARα was detected in 4 µm thick paraffin sections. Slides were deparaffinised and hydrated by passage through a series of xylene, ethanol, and distilled water washes. Heat-induced antigen retrieval in citrate buffer pH6 was performed (120 °C, 15 min, Histos device). The samples were pre-treated with PolyDetector Peroxidase Blocker (Bio SB, part of the detection kit) for 5 min, samples were incubated for 30 min with ProteinBlock (Dako, Glostrup, Denmark) and then incubated with PPARα primary antibody (GeneTex, Hsinchu, Taiwan, cat. no. GTX28934) at dilution 1:100 for 1 h at RT. The reaction was visualised by Mouse/Rabbit PolyDetector DAB HRP Brown kit (Bio SB, Santa Barbara, USA, cat. no. BSB 0205). Tris buffer with TWEEN 20 (pH 7.6) was used for washing between the different steps. Nuclei were counterstained with haematoxylin. After washing in tap water, the samples were dehydrated and cover slipped. 

Stained samples were semi-quantitatively evaluated twice at different times. Evaluation of staining intensity was performed as following: 0 for negative tissue, 1 for a weak signal, 2 for a moderate signal, and 3 for a strong signal. Additionally, for overall staining intensity of the samples, the crypt and epithelial surface areas were evaluated separately for normal colon tissue samples.

### 2.8. Statistical Evaluation

Results obtained from proliferation assay and In-Cell ELISA were evaluated by one-sample *t*-tests. The % of cells with nuclear positivity of PPARα was evaluated by Fisher’s exact test. The differences in PPARα staining intensities between normal and tumour tissues as well as between crypt and surface epithelium in normal colon were evaluated by the Wilcox test. The differences in immunostaining among tumour grades were evaluated by the Kruskal–Wallis test. The lipid content in control and treated cells was evaluated using Student’s *t*-tests. All calculations were performed by GraphPad Prism 8 (San Diego, USA) at the *p* < 0.05 level of significance. Statistically significant differences are marked with an asterisk (*) directly in graphs: * *p* ≤ 0.05, ** *p* ≤ 0.01, *** *p* ≤ 0.001, and **** *p* ≤ 0.0001.

## 3. Results

### 3.1. Expression and Nuclear Localisation of PPARα in Undifferentiated and Differentiated Intestinal Cells

**Figure 1:**
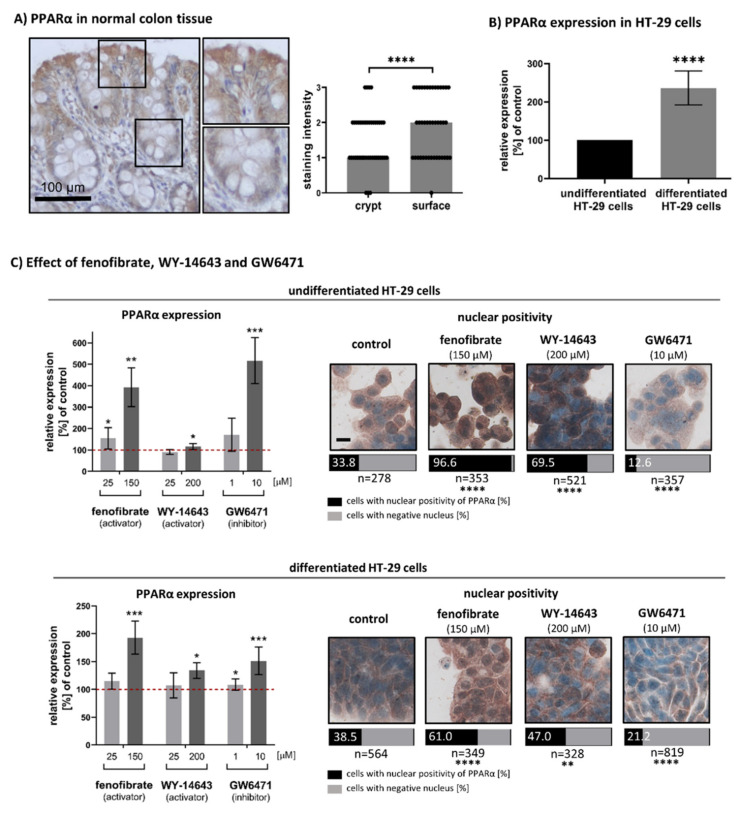
Expression and nuclear localisation of PPARα in undifferentiated and differentiated intestinal cells: (**A**) Comparison of immunostaining intensity in crypt area (undifferentiated cells) and surface epithelium (differentiated cells). The immunohistochemical (IHC) profile is shown as medians of staining intensities of all samples. Staining intensities were evaluated semi-quantitatively: negative (0), weak (1), moderate (2) and strong (3). The results were evaluated by Wilcox test, *n* = 37. Magnification 200x, details 400x, antibody signal brown, nuclei blue. (**B**) Relative change of PPARα expression between undifferentiated and differentiated HT-29 cells measured by In Cell-ELISA. Black columns represent controls, undifferentiated or differentiated cells (100%). Results are shown as mean ± SD (*n* = 6) and evaluated by one sample *t*-test. (**C**) Effect of PPARα activators (fenofibrate and WY-14643) and PPARα inhibitor (GW6471) on PPARα expression and subcellular localisation in HT-29 cells. Relative expression of PPARα in comparison to control was measured by In-Cell ELISA. Results are shown as the mean ± SD (*n* = 6) and evaluated by one-sample *t*-test. The red dotted lines represent control cells: DMSO treated undifferentiated or differentiated cells (100%). Nuclear positivity of PPARα was evaluated as the % of cells with nuclear PPARα positivity from 5 fields of vision. The results were evaluated by Fisher’s exact test. Fenofibrate and WY-14643 favoured nuclear positivity of PPARα, whereas GW6471 retained PPARα in cell cytoplasm. All microphotographs are in the same magnification (400x); the black line represents 10 μm; brown - antibody signal; blue -nuclei. Statistically significant results in comparison to control cells are marked directly in the graphs: * *p* ≤ 0.05, ** *p* ≤ 0.01, *** *p* ≤ 0.001, **** *p* ≤ 0.0001.

In colon tissue sections, the surface epithelium consists of differentiated cells whereas undifferentiated cells are located in crypts. We found a statistically significant increase in PPARα expression in differentiated cells in comparison to undifferentiated ones (*n* = 37, *p* < 0.0001). The median of IHC staining intensity for the crypt area was 1 (weak staining), whereas the median of IHC staining intensity for surface epithelium was 2 (moderate staining). For results, see Figure 1A.

HT-29 cells represent an undifferentiated phenotype when grown in normal culture conditions. They can be differentiated in vitro under experimental culture conditions (incubation with 5 mM sodium butyrate for 72 h) [34]. In differentiated HT-29 cells, we found a 2.36-fold higher expression of PPARα in comparison to undifferentiated ones (*p* < 0.0001). We also detected slightly higher nuclear positivity of PPARα in differentiated cells (33.8% vs. 38.5%), but this difference was non-significant (*p* = 0.1974).

In next experiment, the PPARα activators (fenofibrate and WY-14643) and PPARα inhibitor (GW6471) were administrated to undifferentiated and differentiated (pre-treated with sodium butyrate) HT-29 cells. Fenofibrate, WY-14643 and GW6471 also increased PPARα expression itself, and this effect was more pronounced in undifferentiated HT-29 cells treated with fenofibrate and GW6471. Administration of 150 μM fenofibrate led to a 3.93-fold increase of PPARα expression (*p* = 0.0023) in undifferentiated cells and a 1.92-fold increase (*p* = 0.0006) in differentiated cells. Administration of 200 μM WY-14643 led to weaker but still significant increase: 1.16-fold for undifferentiated cells (*p* = 0.0203) and 1.34-fold for differentiated cells (*p* = 0.0019). Administration of GW6471 increased the PPARα expression 5.17-fold in undifferentiated cells (*p* = 0.0002) and 1.51-fold increased (*p* = 0.0006) in differentiated cells. For results, see Figure 1C.

We also explored the subcellular localisation of the receptor of interest. The fenofibrate, WY-14643 and GW6471 treatment affected nuclear localisation of PPARα receptor in both undifferentiated and differentiated HT-29 cells. As expected, treatment with 150 μM fenofibrate and 200 μM WY-14643 led to a significant increase in nuclear positivity of PPARα in comparison to control cells (*p* < 0.0001 for both compounds in undifferentiated cells and with *p* < 0.0001 for fenofibrate and *p* = 0.0079 for WY-1463 in differentiated cells). Contrary to PPARα activators, GW6471 caused a significant decrease in nuclear positivity of PPARα in comparison to control cells as expected (*p* < 0.0001 for undifferentiated and differentiated cells).

### 3.2. Effect of Fenofibrate, WY-14643 and GW6471 on the Cell Proliferation Activity of HT-29

**Figure 2:**
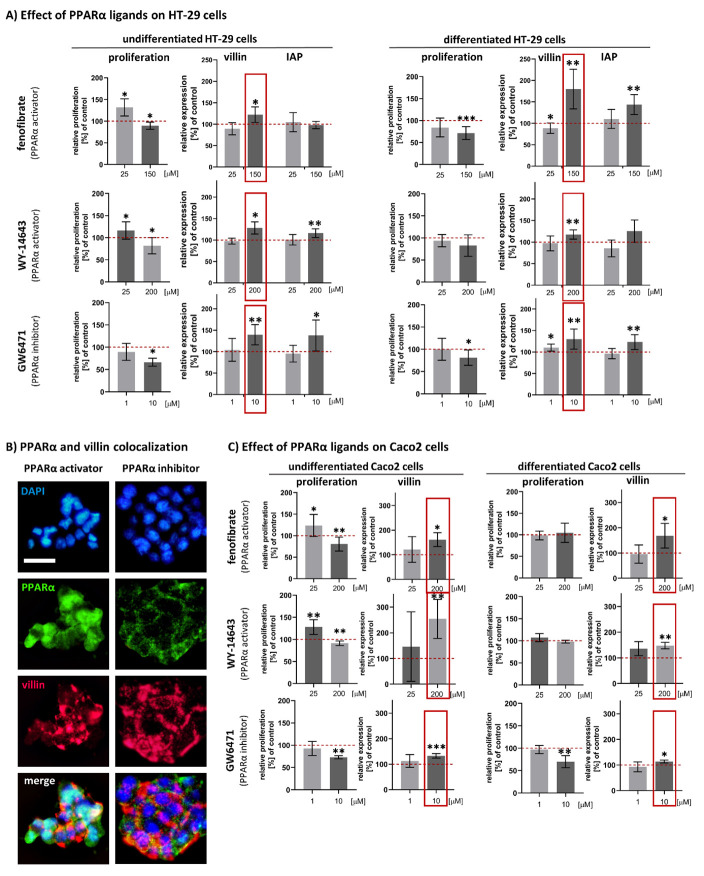
Effects of PPARα activators (fenofibrate and WY-14643) and PPARα inhibitor (GW6471) on cell proliferation and differentiation: (**A**) The effect of PPARα ligands on undifferentiated and sodium butyrate differentiated HT-29 cells. Relative cell proliferation measured by WST-1 assay. Results are shown as the mean ± SD (*n* = 9). Relative expression of villin and intestinal alkaline phosphatase (IAP) in comparison to the control was measured by In-Cell ELISA. Results are shown as the mean ± SD (*n* = 6). The red dotted lines represent control cells: DMSO-treated undifferentiated or differentiated cells (100%). (**B**) Co-localisation of PPARα and villin expression in HT-29 cells. The cells were treated with 150 μM fenofibrate (PPARα activator) and 10 μM GW6471 (PPARα inhibitor). Fenofibrate treatment favoured nuclear localisation of PPARα, whereas GW6471 treatment retained PPARα in cytoplasm. The villin expression was independent of subcellular localisation of PPARα. All microphotographs are in the same magnification (400x); the white line represents 10 μm. (**C**) Effect of PPARα ligands on undifferentiated and spontaneously differentiated Caco2 cells. Relative cell proliferation measured by WST-1 assay. Results are shown as the mean ± SD (*n* = 9). Relative expression of villin in comparison to the control was measured by In-Cell ELISA. Results are shown as the mean ± SD (*n* = 6). The red dotted line represents control cells: DMSO-treated undifferentiated or differentiated cells (100%). Note the increase in villin expression in both used cell lines after PPARα treatment (red rectangles). Statistically significant results in comparison to control cells are marked by * *p* ≤ 0.05, ** *p* ≤ 0.01, *** *p* ≤ 0.001.

The effects of fenofibrate, WY-14643 and GW6471 treatment on cell proliferation for undifferentiated and differentiated HT-29 are summarised in Figure 2A.

In undifferentiated HT-29 cells, lower concentration (25 μM) of PPARα activators fenofibrate and WY-14643 showed an increase in cell proliferation: 131.70 ± 19.71% of control (*p* = 0.0228) for fenofibrate and 116.4 ± 19.67% of control (*p* = 0.0366) for WY-14643. The higher concentrations of PPARα activators (150 μM fenofibrate and 200 μM WY-14643) showed a significant decrease in proliferation activity to 89.41 ± 8.28% of the control (*p* = 0.0259) and 81.81 ± 18.87% of the control (*p* = 0.0384), respectively. Administration of PPARα inhibitor GW6471 showed a concentration-dependent decrease in cell proliferation activity: 89.57 ± 19.18% of the control for 1 μM GW6471 (non-significant, *p* = 0.2403) and 66.49 ± 8.87% of the control for 10 μM GW6471 (*p* < 0.0001). 

In differentiated HT-29 cells, we found a concentration-dependent decrease in proliferation activity after treatment with all compounds. The obtained results (as a % of the control) were: 84.31 ± 21.40% for 25 μM fenofibrate (*p* = 0.1005) and 71.61 ± 14.78% for 150 μM fenofibrate (*p* = 0.0004), 93.85 ± 13.69% (*p* = 0.2448) for 25 μM WY-14643 and 82.90 ± 24.23% (*p* = 0.1444) for 200 μM WY-14643 (*p* = 0.1441), and 100.10 ± 24.66% for 1 μM GW6471 (*p* = 0.9951) and 81.32 ± 17.26% (*p* = 0.0118) for 10 μM GW6471.

### 3.3. Effects of Fenofibrate, WY-14643 and GW6471 on Expression of Intestinal Differentiation Markers (Villin and IAP) in HT-29 Cells

The effects of fenofibrate, WY-14643 and GW6471 on expression of villin and IAP in HT-29 cells was dose-dependent. For the results, see Figure 2A. In general, the treatment with higher concentrations (concentrations decreasing cell proliferation response) of PPARα activators led to a significant increase in villin expression; the increase in IAP expression was less convincing. Fenofibrate treatment increased the villin expression 1.22-fold (*p* = 0.0100) in undifferentiated cells and 1.80-fold (*p* = 0.0019) in differentiated cells. The IAP expression was unchanged in undifferentiated cells, and there was a 1.52-fold increased (*p* = 0.0012) in differentiated cells. WY-14643 increased the villin expression 1.17-fold (*p* = 0.0099) in undifferentiated cells and 1.34-fold (*p* = 0.0019) in differentiated cells. The IAP expression was 1.16-fold increased (*p* = 0.0029) in undifferentiated cells and 1.25-fold increased (*p* = 0.1436) in differentiated cells. 

Surprisingly, the administration of PPARα inhibitor GW6471 showed a very similar pattern of changes in the expression of proteins of interest as PPARα activators. A significant increase in the expression of villin and IAP was clearly apparent after treatment with higher (10 μM) GW6471 concentrations. The villin expression was 1.40-fold higher (*p* = 0.0020) in undifferentiated cells and 1.30-fold higher (*p* = 0.0087) in differentiated cells. The IAP expression increased by 1.38-fold (*p* = 0.0211) in undifferentiated cells and 1.23-fold (*p* = 0.0067) in differentiated cells.

In addition to the ICE results, co-localisation of PPARα and villin expression using multiplex immunofluorescent staining confirmed that villin expression was not dependent on subcellular localisation of PPARα, neither PPARα activation (fenofibrate) nor inhibition (GW6471). For the results, see Figure 2B.

### 3.4. Confirmation of the Effect of Fenofibrate, WY-14643 and GW6471 on Cell Proliferation Activity and Villin Expression in Caco2 Cell Line

To confirm that PPARα activators as well as PPARα inhibitor led to the same result in terms of an increase in villin expression, we also conducted the experiments with the Caco2 cell line. To obtain differentiated Caco2, we used the post-confluent growth for 14 days. Thus, in this case, the differentiation phenotype was not induced by the addition of any compound. 

In undifferentiated Caco2 cells, the proliferation response resembled the trends observed in HT-29 cells. The lower concentration (25 μM) of PPARα activators led to significant increase in cell proliferation: 123.7 ± 25.62% of the control for fenofibrate and 128.0 ± 14.93% of the control for WY-14643 (*p* = 0.0498 and *p* = 0.0058). Higher concentrations (200 μM) of PPARα activators led to a significant decrease in cell proliferation to 80.59 ± 16.15% of the control for fenofibrate and 91.35 ± 5.162% of the control for WY-14643 (*p* = 0.0069 and *p* = 0.0093). Administration of PPARα inhibitor (GW6471) in a higher concentration (10 μM) led to a significant decrease in cell proliferation (*p* = 0.0002). Used compound did not significantly affect proliferation of differentiated Caco2 cells in comparison to untreated differentiated cells. Only the decrease in proliferation caused by the 10 μM GW6471 was significant (70.1 ± 11.86% of the control, *p* = 0.0016). 

The villin expression in Caco2 cells showed the same patterns as the HT-29. In undifferentiated cells, administration of PPARα activators in the concentration of 200 μM increased the expression of villin 1.61-fold for fenofibrate and 2.54-fold for WY-14643 (*p* = 0.0226 and *p* = 0.0026). Treatment with 10 μM GW6471 led to a 1.32-fold increase in villin expression (*p* = 0.0002). The increase in villin expression was also observed in differentiated Caco2 cells treated with fenofibrate, WY-14643 and GW6471. The increase was 1.68-fold, 1.37-fold and 1.13-fold, respectively, (*p* = 0.0357, *p* = 0.0251 and *p* = 0.0187).

### 3.5. Effects of Fenofibrate, WY-14643 and GW6471 on Lipid Content

**Figure 3:**
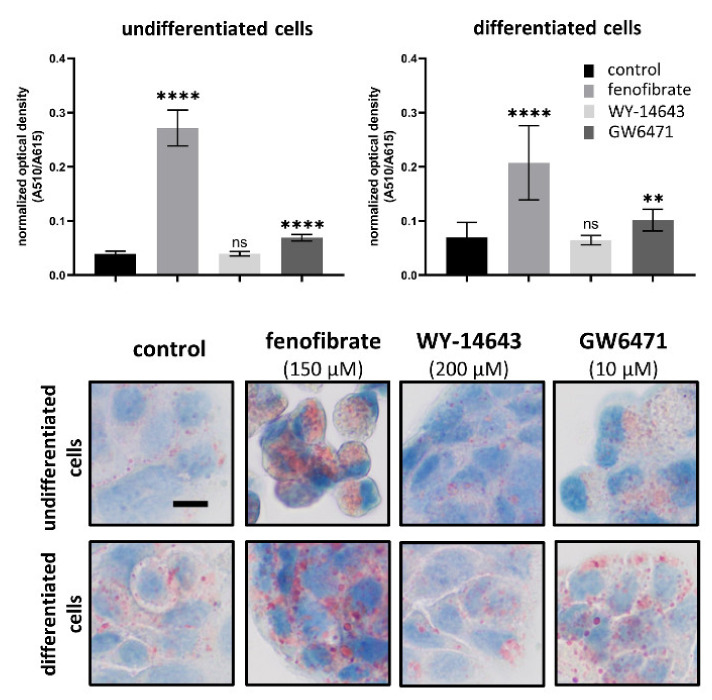
Lipid content in undifferentiated and differentiated HT-29 cells after treatment with PPARα activators (fenofibrate and WY-14643) and PPARα inhibitor (GW6471). The used concentrations were 150 μM for fenofibrate, 200 μM for WY-14643 and 10 μM for GW6471. Lipid content was quantified as absorbance obtained after Oil Red O staining (A510) normalised to Janus green whole-cell staining (A615). Results are shown as the mean ± SD (*n* = 12) and evaluated by the Student’s *t*-test. Statistically significant results in comparison to control cells are marked by ** *p* ≤ 0.01 and **** *p* ≤ 0.0001. All microphotographs are at the same magnification (400x); the black line represents 10 μm; red - lipid droplets; nuclei -blue.

In untreated (control) cells, we observed significant accumulation of lipids in differentiated HT-29 cells in comparison to undifferentiated ones (*p* = 0.0015). The lipid content in differentiated HT-29 cells was twofold higher than in undifferentiated cells. Treatment with 150 μM fenofibrate led to a strongly significant increase in lipid accumulation in both undifferentiated and differentiated cells in comparison to the controls (*p* < 0.0001 for both undifferentiated and differentiated cells). Treatment with 10 μM GW6471 also led to lipid accumulation to a lesser extent than fenofibrate treatment, but the differences between GW6471 treated and control cells were significant (*p* < 0.0001 for undifferentiated cells, *p* = 0.0054 for differentiated cells). Contrary to lipid accumulation after fenofibrate and GW6471 treatment, administration of WY-14643 had no effect on the lipid content. For the results, see Figure 3.

### 3.6. Comparison of PPARα in Tumour and Adjacent Normal Tissue Samples

**Figure 4:**
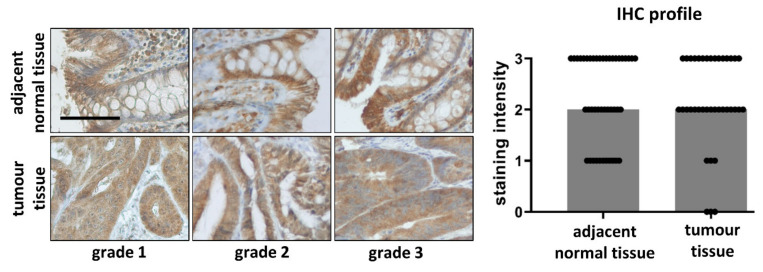
Expression of PPARα in colorectal carcinoma and adjacent normal tissues. Representative microphotographs of grade 1, grade 2 and grade 3 tumours and adjacent normal tissue samples from the same patient. The immunohistochemical (IHC) profile is shown as the medians of staining intensities of all samples. Because there were no differences between tumours and adjacent normal tissues in grade 1, grade 2 and grade 3 tumours, all results for IHC staining are represented together (*n* = 37). Columns represent medians of immunostaining intensities; each dot represents one patient. Magnification 100x; black line represents 100 μm; brown - antibody signal; blue - nuclei.

We found no difference between PPARα immunostaining intensities between tumour and adjacent normal tissue samples (*p* = 0.6182, *n* = 37). We also found no differences in IHC staining intensities between tumours and adjacent normal tissue samples when we analysed each tumour grade separately with *p* = 0.3750, *p* = 0.2323 and *p* = 0.6875 for grade 1, grade 2 and grade 3, respectively. Moreover, there were no significant differences in immunostaining intensities of grade 1, grade 2 and grade 3 tumours (*p* = 0.3924). The decrease in expression of PPARα in carcinoma samples in comparison to normal tissue was detected in 15/37 patients (i.e., 40.5%), the increase in 14/37 (37.8%) patients and 8/37 (21.6%) patients samples showed the same staining intensity for normal and tumour tissue samples. Moreover, we found no differences in PPARα expression in tumours between males and females (*p* = 0.6875) as well as when we evaluated differences between tumours and adjacent normal tissues for males and females separately with *p* = 0.4112 and *p* = 0.5870. Because no differences among tumour grades were detected, the immunostaining intensities in Figure 4 were grouped and represented all together. The columns show medians of staining intensity, each dot represents one patient (*n* = 37). The results are accompanied by representative microphotographs of grade 1, grade 2 and grade 3 tumours and adjacent normal tissues from the same patient.

## 4. Discussion

PPARα is involved in various cellular functions including differentiation of various cell types. The aim of this study was to investigate the possible role of PPARα in intestinal cell differentiation using in vitro differentiated HT-29 and Caco2 cells and tissue samples of normal epithelium as well as colorectal carcinoma. 

Our results showed an increase in PPARα expression in differentiated cells of both colon tissue samples as well as in vitro differentiated HT-29 cells. The increase in PPARα expression in differentiated HT-29 cells resembled the expression of villin described in our previous study [34]. In differentiated HT-29 cells, we detected 2.36-fold and 2.15-fold higher levels of PPARα and villin, respectively. An Increase in PPARα expression in differentiated intestinal cells has previously been described. It was shown that PPARα expression is stronger in the apical part of villi in mouse small intestine [30] as well as in in vitro differentiated Caco2 cell line which was accompanied by an increase in the nuclear positivity of PPARα [32]. Although the expression of PPARα increased, the nuclear positivity remained comparable between undifferentiated and differentiated HT-29 cells in our experiment.

The first prerequisite for the regulation of gene expression is the nuclear localisation of the receptor. It has been shown that PPARα shuffle between cytoplasm and nucleus [35], and nuclear localisation is favoured by ligand binding. Our results confirmed an increase in nuclear subcellular localisation of PPARα after administration of PPARα activators. Contrary to this, PPARα inhibitor reduced PPARα nuclear positivity as expected. This phenomenon was observable regardless of the differentiation status of HT-29 cells; however, it was more pronounced in undifferentiated cells.

Both PPARα activators (fenofibrate and WY-14643) as well as PPARα inhibitor (GW6471) affected cell proliferation activity. Cell treatment with 150 μM fenofibrate, 200 μM WY-14643 and 10 μM GW6471 led to significant decreases in cell proliferation of HT-29 cells, regardless of the differentiation status. The significant decreases in cell proliferation after treatment with 200 μM fenofibrate, WY-14643 and 10 μM GW6471 were detected also in undifferentiated Caco2 cells. The decrease in cell proliferation is in accordance with previous studies performed in various human cell types treated with fenofibrate [11,12,13,14,36,37,38,39,40,41,42,43], WY-14643 [44,45] or GW6471 [46,47,48]. An increase in cell proliferation, observed in undifferentiated cells after treatment with lower concentration of fibrates, was also previously described [17,18].

The undifferentiated and differentiated HT-29 cells showed similar response to treatment with PPARα activator and inhibitor regardless of their differentiation status. The administration of PPARα activators led to an increase in villin and IAP expression suggested the role of PPARα activation in intestine cell differentiation. Surprisingly, the administration of PPARα inhibitor led to the same results. Moreover, we proved that HT-29 cells expressed villin independently on PPARα subcellular localisation. The same trend in villin expression was also observed in Caco2 cell line. Although it may seem at first glance that PPARα may play a role in differentiation of intestinal cells due to the fact of its higher expression in differentiated cells in comparison to undifferentiated ones, our data indicated intestinal cell differentiation was PPARα independent. We suppose that the increase in differentiation markers after fenofibrate, WY-14643 and GW6471 was related to the decrease in cell proliferation rather than direct PPARα activation or inhibition.

According to available literature, villin functions are regulated via PI3K/Akt-mediated signalling, because association of villin with phosphatidylinositol(4,5)-bisphosphate (PIP2) enhances its actin bundling function and, thus, formation of brush border [49,50,51]. PI3K phosphorylates PIP2 to phosphatidylinositol(3,4,5)-trisphosphate (PIP3). An increase in expression of markers of differentiation was observed after concentration of fenofibrate and GW6471 that inhibit cell proliferation activity, which could be mediated via the PI3K/Akt pathway. It has been shown that GW6471 decreases the expression of PI3K in cells of head and neck paragangliomas [46]. The same effect, a decrease in PI3K, has been observed in human gastric cancer cell lines after fenofibrate treatment [37]. A decrease in PI3K could be associated with PIP2 accumulation and thereby the actin bundling function of villin. In colorectal carcinoma cells HCT-116, inhibition of PI3K has led to an increase in alkaline phosphatase activity [52]. Moreover, it has been shown that fenofibrate suppresses growth via a decrease in phosphorylation of Akt, and this effect is PPARα independent in hepatocellular carcinoma cells [36] as well as in angiosarcoma cells [38]. However, if upstream molecules, such as PI3K, are also affected, they have not been described yet. The observed effect of WY-14643 on villin expression in our study may also be PPARα-independent. Except involvement of the PI3K pathway, intestinal cell differentiation has also been associated with activation of p38 MAPK [53,54], and it has been shown that WY-14643 induces phosphorylation of this protein [55,56,57]. 

PPARα is known as a lipid sensor. PPAR-α controls the expression of numerous genes related to lipid metabolism, including genes involved in mitochondrial β-oxidation, peroxisomal β-oxidation, fatty acid uptake and binding and lipoprotein assembly and transport [5]. It has been shown that HT-29 cells cultured with sodium butyrate increases the amount of lipid droplets [58,59]. We also observed an increase in lipid droplet accumulation in sodium butyrate differentiated HT-29 cells. Thus, it could seem to be associated with differentiation of intestinal cell. However, understanding of this phenomenon is elusive. Lipid droplet accumulation is a well-known hallmark of cancer, including colorectal carcinoma, and it has been associated with cancer proliferation and aggressiveness [48,60,61,62,63,64]. Moreover, it has been shown that stimulation of lipid droplet density promoted proliferation in colon cancer cells [65]. The effect of PPARα ligands on lipid droplet accumulation is not clear. Previous studies have shown that although fenofibrate has lowered lipid content in C2C12 myotubes [66], the same compound has caused dose-dependent lipid droplets accumulation in HepG2 cells [67]. GW6471 has decreased lipid droplets in breast cancer cells [48]. We observed enhanced lipid droplet accumulation in cells treated with fenofibrate as well as GW6471. Contrary to this, the second used PPARα activator, WY-14643, had no effect on lipid accumulation. The observed lipid droplet accumulation was not associated with expression of villin and, thus, with intestinal cell differentiation.

Carcinogenesis is the disruption of normal differentiation process. PPARα appears to play a role in carcinogenesis; however, it may act as a tumour suppressor or an oncoprotein [11,12,13,14,15,16,17,18,19,46,47,68,69]. Colorectal carcinoma is the third most common cancer in terms of incidence but the second in terms of mortality [70]. The role of PPARα in colorectal cancer is inconclusive. At the tissue level, Luo et al. described reduced levels of PPARα mRNA in colon cancer from mice. Moreover, activation of PPARα by fenofibrate protected human PPARα transgenic mice from chemical-induced colon cancer [71]. Contradictory results were described by Yaghoubizadeh et al. They detected overexpression of PPARα mRNA in colorectal tumour tissues in comparison to adjacent normal tissues. This was negatively associated with clinico-pathological factors, such as tumour size, grade, TNM stage, metastases, lymphatic invasion and decrease in overall survival [31]. Using immunohistochemical detection of PPARα in human tissue samples, Morinishi et al. described higher PPARα positivity in carcinoma tissues than in normal epithelium. However, PPARα expression was not related to sex, age, lymphatic invasion, venous invasion, lymph node metastasis, depth of invasion and stage [4]. In our tissue sample collection, we detected comparable levels of PPARα in tumours in comparison to adjacent normal tissues. Moreover, there was no relation of PPARα expression in tumours and tumour grades. These observations supported our results obtained for HT-29 and Caco2 cell lines—that differentiation of intestinal cell is PPARα independent. 

## 5. Conclusions

Taken together, our study revealed a significant increase in PPARα expression in differentiated HT-29 cells as well as in normal surface colon epithelium where differentiated cells are localised. Interestingly, we found that both, PPARα activators, fenofibrate and WY-14643 as well as its inhibitor GW6471 regulated proliferation and differentiation of HT-29 cells in vitro in the same way. Both compounds led to a decrease in proliferation accompanied with an increase in expression of villin and IAP. The same trend in villin expression was confirmed in Caco2 cells. Furthermore, villin expression was independent of subcellular localisation of PPARα. Moreover, we found similar levels of PPARα expression in colorectal carcinomas in comparison to adjacent normal epithelium. All these findings support the hypothesis that differentiation of intestinal epithelium is PPARα independent.

## Data Availability

Data is contained within the article or Supplementary Materials. The patient data presented in this study are available in Supplementary File Table S1.

## Supplementary Materials

The following are available online at https://www.mdpi.com/article/10.3390/biomedicines9091255/s1, Table S1: Characteristics of tissue samples used in this study. All patients were Causasians with no anticancer therapy before surgery. c.—colon, T—primary tumour, N—lymph nodes, M—distant metastases, G1—grade 1, G2—grade 2, G3—grade 3; Figure S1: Schematic summarization of experimental procedure. The experimental procedure for undifferentiated cells of both cell lines were following: the cells were seeded, adhered overnight (o.n.), treated with PPARα ligands or DMSO (controls), incubated for 72 h and then the analysis was performed (proliferation assay, In-Cell ELISA, immunofluorescent and immunocytochemical staining). To obtain differentiated cells, the cells were pre-treated with 5mM sodium butyrate (NaBt) for 72 h (HT-29) or growth for 14 days after reaching confluence (Caco2). After differentiation procedure, the medium was changed and the cells were treated with PPARα ligands or DMSO (controls), incubated for 72 h and then the analysis was performed. The cells were seeded on 96-well culture plates or 8-well culture slides, seeding density dependent on the assay and cell line.
