# Supplementary material for: When Activator and Inhibitor of PPARα Do the Same: Consequence for Differentiation of Human Intestinal Cells
Source: Biomedicines. 2021 Sep 17;9(9):1255. doi: 10.3390/biomedicines9091255 (PMC8472525; doi:10.3390/biomedicines9091255)
Supplement: Supplementary file 1 [file biomedicines-09-01255-s001.zip › Supplementary file - Figure S1.pdf]

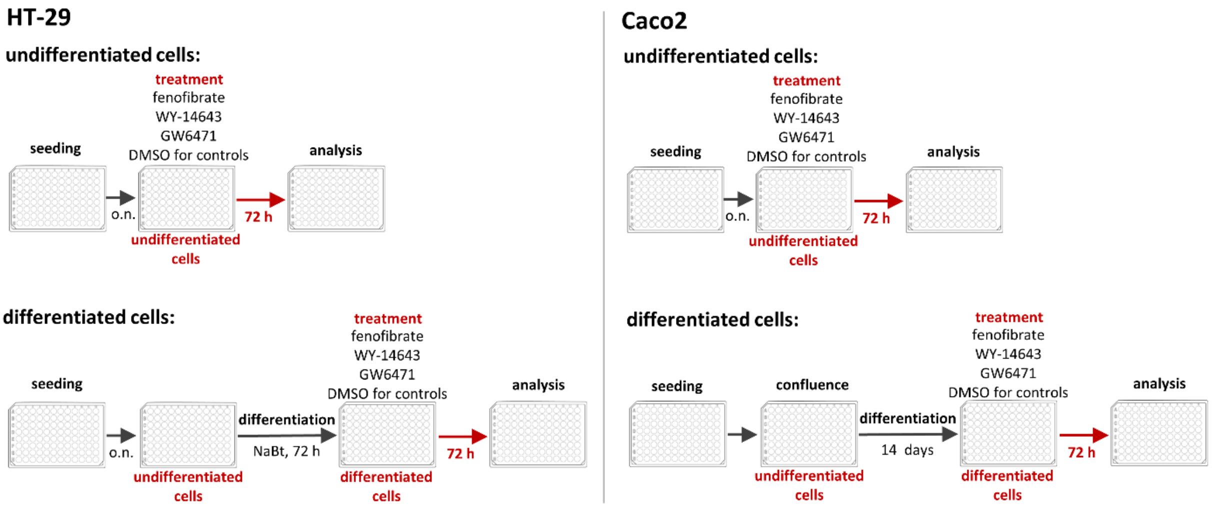

Figure S1: **Schematic summarization of experimental procedure.** The experimental procedure for undifferentiated cells of both cell lines were following: the cells were seeded, adhered overnight (o.n.), treated with PPARα ligands or DMSO (controls), incubated for 72 h and then the analysis was performed (proliferation assay, In-Cell ELISA, immunofluorescent and immunocytochemical staining). To obtain differentiated cells, the cells were pre-treated with 5mM sodium butyrate (NaBt) for 72 h (HT-29) or growth for 14 days after reaching confluence (Caco2). After differentiation procedure, the medium was changed and the cells were treated with PPARα ligands or DMSO (controls), incubated for 72 h and then the analysis was performed. The cells were seeded on 96-well culture plates or 8-well culture slides, seeding density dependent on the assay and cell line.
