# Supplementary material for: When Activator and Inhibitor of PPARα Do the Same: Consequence for Differentiation of Human Intestinal Cells
Source: Biomedicines. 2021 Sep 17;9(9):1255. doi: 10.3390/biomedicines9091255 (PMC8472525; doi:10.3390/biomedicines9091255)
Supplement: Supplementary file 1 [file biomedicines-09-01255-s001.zip › Supplementary file - Table S1.pdf]

Table S1: **Characteristics of tissue samples used in this study**. All patients were Causasians with no anticancer therapy before surgery. c. – colon, T – primary tumour, N – lymph nodes, M – distant metastases, G1 – grade 1, G2 – grade 2, G3 – grade 3

no.	sex	age	diagnosis	localization	TNM staging			grading
					T	N	M	
1	male	71	adenocarcinoma	c. sigmoideum	T3	N0	M0	G2
2	female	66	adenocarcinoma	c. sigmoideum	T3	N0	M0	G2
3	male	66	adenocarcinoma	c. sigmoideum	T3	N0	M0	G3
4	male	51	adenocarcinoma	c. sigmoideum	T2	N0	M0	G2
5	female	63	adenocarcinoma	c. descendens, rectum	T3	N1	M1	G2
6	male	72	adenocarcinoma	c. descendens, rectum	T2	N0	M0	G2
7	male	70	adenocarcinoma	c. descendens, rectum	T3	N0	M0	G2
8	female	74	adenocarcinoma	c. descendens, rectum	T2	N0	M0	G2
9	male	76	adenocarcinoma	c. sigmoideum	T4a	N0	M0	G2
10	female	50	adenocarcinoma	c. sigmoideum	T3	N0	M0	G1
11	male	72	adenocarcinoma	c. sigmoideum	T3	N1a	M0	G2
12	male	60	adenocarcinoma	c. sigmoideum	T4	N2a	M1b	G1
13	female	50	adenocarcinoma	c. sigmoideum	T3	N2b	M1b	G3
14	male	77	adenocarcinoma	c. sigmoideum	T3	N0	M0	G3
15	male	69	adenocarcinoma	c. ascendens	T3	N2b	M0	G2
16	male	64	adenocarcinoma	c. sigmoideum	T2	Nx	Mx	G1
17	male	34	adenocarcinoma	c. sigmoideum	T2	Nx	M0	G1
18	female	79	adenocarcinoma	c. ascendens	T2	Nx	M0	G1
19	male	83	adenocarcinoma	c. descendens, rectum	T2	Nx	M0	G1
20	male	77	adenocarcinoma	c. sigmoideum	T3	N1	Mx	G2
21	male	70	adenocarcinoma	c. sigmoideum	T4a	N0	M1a	G2
22	female	75	adenocarcinoma	c. sigmoideum	T3	Nx	Mx	G1
23	male	57	adenocarcinoma	c. sigmoideum	T2	Nx	Mx	G2
24	male	60	adenocarcinoma	c. sigmoideum	T3	N0	M0	G1
25	male	71	adenocarcinoma	c. sigmoideum	T2	N0	M0	G2
26	male	54	adenocarcinoma	c. sigmoideum	T2	N0	M0	G3
27	female	68	adenocarcinoma	c. sigmoideum	T2	N0	M0	G2
28	male	60	adenocarcinoma	c. sigmoideum	T4a	N2a	M0	G2
29	male	47	adenocarcinoma	c. sigmoideum	T3	N0	M0	G2
30	male	63	adenocarcinoma	c. descendens, rectum	T3	N2b	M0	G3
31	male	86	adenocarcinoma	c. descendens, rectum	T3	N1b	M0	G2
32	female	70	adenocarcinoma	c. descendens, rectum	T3	N2a	M0	G3
33	female	73	adenocarcinoma	c. descendens, rectum	T2	N0	M0	G2
34	female	84	adenocarcinoma	c. descendens, rectum	T3	N0	M0	G1
35	male	76	adenocarcinoma	c. sigmoideum	T3	N0	M0	G3
36	male	55	adenocarcinoma	c. sigmoideum	T3	N2b	M0	G2
37	male	69	adenocarcinoma	c. sigmoideum	T3	N0	M0	G3
